# Vocal Outcome Following Thyroidectomy for Differentiated Thyroid Carcinoma

**DOI:** 10.3390/jcm13247576

**Published:** 2024-12-12

**Authors:** Maria Silvia Rosa, Valeria Dell’Era, Massimo Campagnoli, Silvia Campini, Ottavia Barozza, Giovanni Borello, Massimiliano Garzaro, Paolo Aluffi Valletti

**Affiliations:** 1 ENT Division, Health Science Department, School of Medicine, 28100 Novara, Italy; 2 Department of Otorhinolaryngology, Ss. Trinità Hospital, 28021 Borgomanero, Italy

**Keywords:** differentiated thyroid carcinoma (DTC), thyroid surgery, vocal outcomes, Acoustic Voice Quality Index (AVQI), thyroid, voice

## Abstract

**Background:** Voice alteration is a recognized complication of thyroid surgery, impacting the quality of life and communication for affected individuals. In this prospective observational study, the Acoustic Voice Quality Index (AVQI) was employed to assess vocal outcomes after thyroidectomy. **Method:** Between February 2018 and August 2022, 224 patients underwent Thyroid surgery in our department, of which 74 with differentiated thyroid carcinoma (DTC) were enrolled in accordance with the inclusion criteria. Endoscopic findings and AVQI scores were evaluated before and after surgery (voice analysis was conducted using the Praat software program version 6.0.33). **Results:** Vocal fold impairment was present in 6.76% of patients after surgery (T1), with full recovery within 3 months. During preadmission evaluation, an AVQI score > 2.35 indicating hoarseness was present in 37 patients (despite normal vocal cord motility). Of these, 25 (67.57%), 26 (70.27%), and 24 (17.76%) maintained this trend at T1, T2, and T3, respectively. No significant variation in mean AVQI values was observed based on gender, age, and central neck dissection. **Conclusion:** AVQI values did not show significant variations comparing pre- and postoperative values. Thyroid surgery for DTC performed by experienced surgeons does not seem to impact significantly on patients’ voice quality.

## 1. Introduction

Voice alteration remains a significant complication of thyroid surgery due to recurrent laryngeal nerve (RLN) injury. During Thyroid surgery, iatrogenic dysphonia caused by vocal fold injury can result not only from thyroidectomy (with reported rates ranging from 0.85% to 8.5%) but also from intubation, which is not uncommon (with estimates ranging widely from 2.3% to 84%) [1]. Regarding thyroid surgery, the most common cause of dysphonia is a lesion or paralysis of the RLN. The incidence of temporary vocal cord dysfunction (resolved within 6 months of surgery) is approximately 9.8%, ranging from 1.4% to 38.4%, while permanent dysfunction occurs in about 2.3%, ranging from 0% to 18.6% [2].

It is also noted that dysphonia can result from superior laryngeal nerve (SLN) injury, primarily affecting professional voice users as it impairs their ability to produce higher vocal registers and decreases vocal projection. The reported incidence of injury to the external branch of the SLN during thyroidectomies is as high as 58%. However, isolated injury of the external branch can be challenging to detect due to minor symptoms and variability in clinical presentation. Dysphonia related to strap muscles damage has been reported in the literature [3].

Possible causes of iatrogenic RLN damage during thyroidectomy could be accidental thermic injury related to the cauterizing system, stretching, or strong compression of the nerve. Rarely does a nerve transaction occur during surgery [4]. All vocal disorders, both functional and iatrogenic, can significantly impact social and occupational functioning, affecting quality of life (QoL). Affected patients often experience social isolation, depression, anxiety, missed work, lost wages, and lifestyle changes. Studies of voice disorders indicate QoL implications and work productivity losses comparable to those of patients with asthma, acute coronary syndrome, depression, and COPD.

About 12,000 new diagnoses of differentiated thyroid carcinoma (DTC) are made yearly in Italy, with DTC being the most frequent endocrine neoplasm.

Its incidence is constantly increasing worldwide due to the ever earlier diagnosis of suspicious thyroid nodules [1,2,3,5]. The surgical treatment of thyroid carcinomas mainly consists of lobectomy or total thyroidectomy, with or without central compartment nodal dissection.

Most cases of RLN paralysis recover without intervention, and invasive therapy should be avoided for at least six months, except in cases of emergency respiratory issues. This complication results in vocal impairment, with temporary or permanent worsening of voice quality. To mitigate these complications, careful surgical techniques should be employed, and when available, intraoperative neuromonitoring (IONM) is recommended. This technique is increasingly utilized during thyroidectomy and parathyroidectomy, especially by young and experienced surgeons, as described by Christou et al. [6]. It is particularly useful in cases with difficult anatomy due to prior surgery, history of radiation therapy, or in patients affected by large volume tumors/goiters.

In clinical practice, voice quality assessment should be conducted using a multidimensional approach, as suggested by the guidelines of the European Laryngological Society. Evaluation tools include auditory-perceptual judgments, video-laryngostroboscopy, acoustic and aerodynamic analysis, and the patient’s subjective evaluations [2].

The literature contains many validated voice quality evaluation questionnaires such as the patient-reported VQ assessment or the 10-voice handicap index (VHI), but they provide a subjective evaluation of the voice [7].

An objective method to quantify the severity of overall voice quality impairment in concatenated continuous speech and sustained phonation segments is the Acoustic Voice Quality Index (AVQI) [8].

The AVQI is a multivariate construct based on linear regression analysis that combines different acoustic parameters to obtain a single score for estimating overall voice quality. Several validation studies have been conducted in various languages, revealing AVQI as a robust measurement tool with high validity, good diagnostic accuracy, and high responsiveness to voice changes through voice therapy [9]. Given the high diagnostic accuracy of this tool, the purpose of this study is to objectively investigate the vocal outcomes following thyroidectomy for DTC, independently of perceptual and subjective analyses.

Furthermore, AVQI scores were compared with variables such as gender, age, profession, endoscopic findings, and type of surgery, which could impact voice preservation.

The present study aimed to objectively investigate the vocal outcomes following thyroid surgery for DTC, employing the Acoustic Voice Quality Index (AVQI).

The second objective of the present study was to investigate whether gender, age, and surgery extension (central neck dissection—CND) could be considered factors influencing voice outcome after surgery.

## 2. Materials and Methods

A prospective observational study was conducted on 74 patients who underwent thyroidectomy for DTC at the ENT clinic of Maggiore Hospital in Novara between February 2018 and August 2022, with a minimum follow-up of 3 months. The study received ethical approval from the Ethical Committee (CE 133/22) and was conducted under the ethical standards of the Helsinki Declaration.

The inclusion criteria were as follows:

- Age > 18 years old;
- Italian speakers;
- Patients who underwent total thyroidectomy or lobectomy, with or without lymph node dissection, for DTC;
- Patients who provided informed consent.

The exclusion criteria were as follows:

- Presence of previous vocal cord pathologies;
- Patients affected by neurologic diseases;
- Patients affected by RLN paralysis or vocal cord impairment at diagnosis;
- Patients who needed to undergo radicalization of the thyroid gland after the first lobo-isthmectomy surgery.

All patients underwent surgery performed by the same experienced ENT surgeons. Particular attention was paid during surgery to identify and preserve the RLN and the parathyroid gland. Antibiotic prophylaxis was administered.

To preserve RLN, an accurate dissection of the thyroid gland was performed, identifying the anatomical landmarks of the nerve such as the inferior parathyroid glands and Zuckerkandl tubercle. Once identified RLN thyroid gland dissection is completed following the nerve along its course.

Central neck dissection was performed according to ESMO thyroid cancer guidelines [10].

The average hospitalization time was 2.36 days, during which no major complications were registered. AVQI was performed before and after surgery with scheduled follow-up visits.

AVQI scores were obtained through the Praat software program version 6.0.33, which provides the spectrogram and vocaligram of the analyzed vocal segment, calculating different parameters related to the fundamental frequency and perturbations of the vocal signal. These parameters are crucial for detecting vocal fold pathologies, as vocal emission is minimally affected by supraglottic traits, and voice analysis strongly reflects vocal cord functionality [11,12]. The AVQI cut-off value for discriminating between normophonic and hoarse voices for Italian speakers is 2.35 (normophonic if ≤2.35).

Preliminary endoscopic investigations were conducted before surgery (T0) and at discharge. An AVQI evaluation was performed at T0 and 10 days postoperatively (T1) during an outpatient visit. The second follow-up visit was scheduled approximately 30 days (T2) after the operation for vocal analysis. The final evaluation was conducted 90 days after surgery (T3) for all patients and after 6 months for patients who experienced complications. A voice analysis was performed using a microphone connected to a PC running the Praat software program. To minimize background noise differences, analyses were always conducted in the same silent room. Once the equipment was set up, with the microphone placed about 10 cm from the mouth, the patient was asked to read five different sentences, as detailed in Table 1, with English translations provided.

The first sentence involves the production of all the vowels of the Italian language, the second sentence is entirely voiced, the third elicits a hard vocal attack, the fourth sentence includes nasal phonemes, and finally, the last one consists of voiceless occlusive phonemes.

During the second registration, the patient is asked to sustain the vowel “a” at a constant frequency and intensity for at least 5 s. Once the spectrogram of the vowel has been obtained, the central 3 s are selected to exclude long-term perturbations linked to the beginning and end of the sound emission. After the tests, the Acoustic Vocal Quality Index (AVQI) was calculated using the data obtained from them. The cut-off value for discriminating between normophonic and dysphonic voices is 2.35. Fiber optic laryngoscopy was routinely performed during the preoperative investigations and at discharge. During follow-up, this examination was repeated only in case of impairment of laryngeal motility detected at discharge or worsening of voice.

All patients with vocal cord impairment at T1 started personal speech rehabilitation within 15 days of discharge.

The analysis was conducted using SPSS for Windows 29.0 (IBM Corp, Chicago, IL, USA). The normality of continuous variables was verified with the Shapiro–Wilk test (normal for *p* > 0.05). The *t*-test for paired samples and the ANOVA test were utilized for normally distributed data. All results are reported as mean ± standard deviation (SD). Statistical significance was assumed for *p*-values < 0.05.

## 3. Results

A total of 224 total or partial thyroidectomies were performed at the ENT Department of Maggiore Hospital in Novara (Italy) during the examined period. Seventy-four patients affected by differentiated thyroid carcinoma (DTC) met the inclusion criteria.

The mean age at diagnosis was 57 years old, with an age range of 23 to 84 years old. More than two-thirds of the population were female, constituting 68.91% (51 out of 74 patients).

Twenty-three out of 74 patients who underwent surgery for DTC were considered voice professionals, given the significance of their voice in their daily work activities (such as teachers, singers, and other professional speakers).

Characteristics of patients, surgery procedure, staging, and complications are summarized in Table 2.

During the period considered for this study, intraoperative neuromonitoring (IONM) of the laryngeal nerves was not systematically employed during thyroidectomy.

- **Endoscopic findings**

All 74 patients presented with normal chord motility at preoperative evaluation.

After surgery, five patients (6.76%) presented with monolateral vocal fold impairment, which was assessed during video-laryngostroboscopy at T1.

During endoscopic follow-up at T2, two patients presented vocal cord impairment, and no patients had laryngeal motility problems at T3. In the end, all of the five patients with laryngeal motility impairment fully recovered complete laryngeal function within 3 months, with three cases recovering within 1 month after early speech therapy rehabilitation.

- **AVQI analysis**

Regarding the acoustic analysis, we initially investigated the percentage of patients presenting with an AVQI > 2.35, indicating hoarseness. Before surgery, 37 patients (50%) showed values above the cutoff despite normal vocal cord motility. Of these, 25 (67.57%), 26 (70.27%), and 24 (17.76%) maintained this trend at T1, T2 and T3, respectively.

It has been noticed that during follow-up visits, laryngeal motility improvement was not correlated with AVQI values normalization. The data of the five patients affected by vocal cord impairment are presented in Table 3.

**Figure 1:**
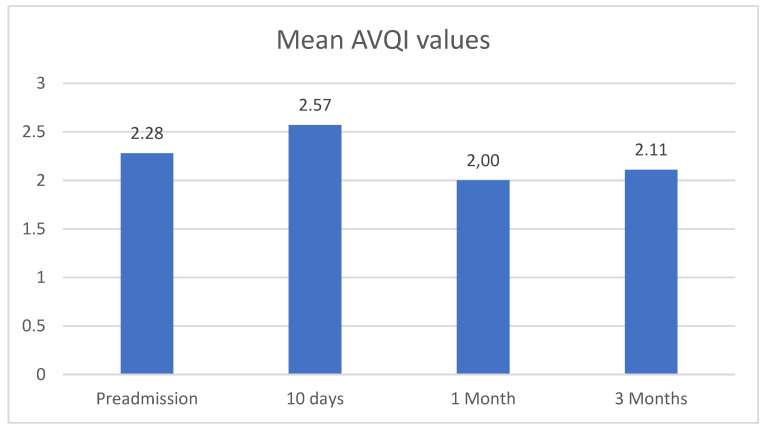
Mean AVQI values at T0, T1, T2, and T3 in the general population.

Comparing pre- and post-operative controls, mean AVQI values did not show significant variations (*p* = 0.68) (Figure 1).

- **Impact of covariates**

Moreover, variations in AVQI values do not appear to be significantly associated with gender, age (≤60 or >60), or central neck dissection.

**Figure 2:**
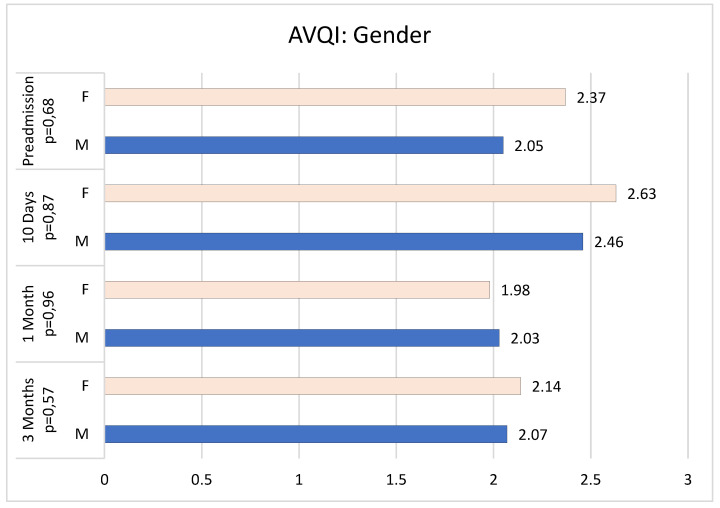
AVQI values at T0, T1, T2, and T3 by gender.

**Figure 3:**
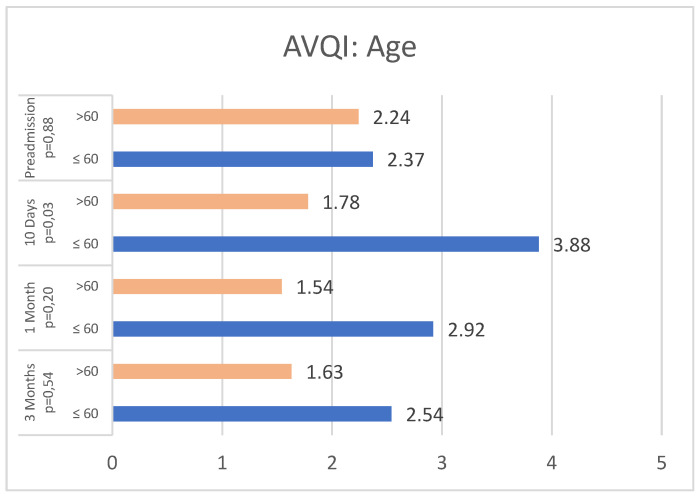
AVQI values at T0, T1, T2, and T3 by age.

In our population, gender does not seem to influence AVQI values before and after surgery (T0 *p* = 0.68; T1 *p* = 0.87; T2 *p* = 0.96; T3 *p* = 0.57) (Figure 2 and Figure 3).

Considering the age groups (≤60 years and >60 years), AVQI values at pre-admission were similar between the two groups (n = 43 and 31, respectively). However, ten days post-surgery, AVQI appears to be statistically lower in older patients (*p* = 0.03), in contrast to the younger group, where AVQI values tended to increase. This trend remains one-month post-surgery, albeit without statistical significance (*p* = 0.20).

**Figure 4:**
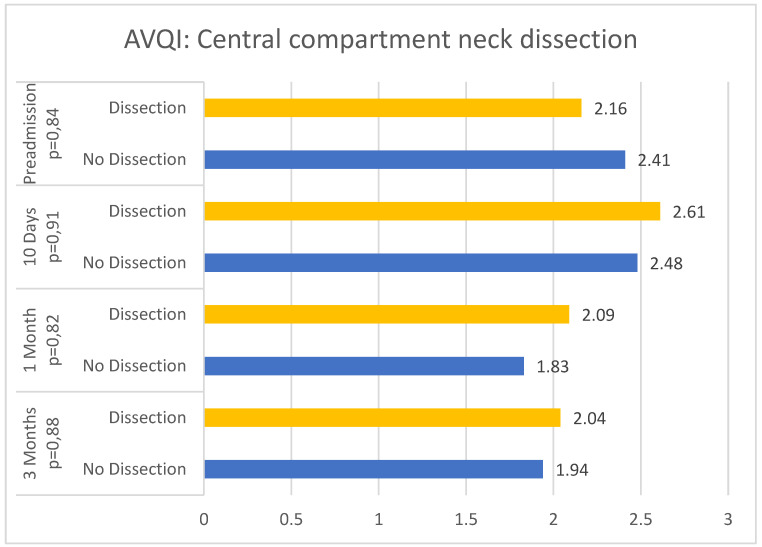
AVQI values at T0, T1, T2, and T3 by central compartment neck dissection.

In the study population, the dissection of the central compartment does not appear to influence postoperative AVQI values (T1 *p* = 0.91; T2 *p* = 0.82; T3 *p* = 0.88) (Figure 4).

Focusing on the five patients with vocal fold impairment at discharge, the presence of confirmed hypomobility after 10 days (T1) is associated, as expected, with an increasing mean value of AVQI (5.21). By T2, it slightly decreased to a value of 4.43, presumably due to the progressive recovery of cord motility and/or compensatory factors. All patients underwent total thyroidectomy. Histological diagnosis was papillary carcinoma in three cases and follicular carcinoma in two. Two of them underwent central neck dissection. In four cases, patients with vocal cord impairment were younger than 60 years of age and presented with large hyperfunctioning nodular goiter (mean maximum dimension of tumoral node 3.45 cm ± 0.84). A very high AVQI score was recorded in the only patient who experienced a postoperative bleeding complication requiring revision surgery (8.62 and 7.75, respectively, at 10 and 30 days, vs. 3.97 preoperative).

## 4. Discussion

The voice represents the fundamental tool for communication between human beings, and its loss or alteration can cause not only physical but also psychological damage due to its impact on the quality of life and social and work activities [9]. Vocal complications following thyroid surgery are a well-known problem in the scientific community. Though often resulting in minor and transient alterations, they can significantly impact social and psychological well-being [13].

Voice dysfunction after thyroidectomy may result from surgical damage to laryngeal nerves or lesions to strap muscles, leading to the impairment of laryngotracheal movement. Damage to the recurrent laryngeal nerve (RLN) is typically recognizable with traditional indirect laryngoscopy and fiber-optic examination. Continuous intraoperative vagus monitoring is a modern technique that can help reduce post-surgical RLN damage, especially in high-risk patients [14]. Damage to the superior laryngeal nerve (SLN) is more challenging to detect and is rarely reported in studies related to post-thyroidectomy voice evaluation [15].

SLN damage can manifest with hoarseness but may also be asymptomatic, particularly if compensation occurs from the contralateral vocal cord [16].

The incidence of SLN damage after thyroid surgery varies in the literature, with reported percentages ranging from 0% to 58%, depending on the authors and surgical techniques employed. Extra-laryngeal muscles also contribute to vocal function, especially in professional singers, and iatrogenic damage to strap muscles or cervical scar shrinkage may cause subtle voice dysfunction even in the absence of visible glottic movement impairment [15].

Various tools are available for voice evaluation, including auditory-perceptual judgments, videolaryngostroboscopy, acoustic and aerodynamic analysis, and subjective evaluations by patients. Acoustic measurements with AVQI provide an objective assessment of voice quality and represent a non-invasive and cost-effective technique for quantifying hoarseness and documenting functional outcomes after surgical and rehabilitation treatments [17].

Despite efforts made, the incidence of temporary vocal cord dysfunction after thyroidectomy reported in the literature ranges from 1.4% to 38.4%, while permanent dysfunction is reported to be about 2.3% [2]. In our study, 6.76% of patients experienced impaired cord motility after surgery but fully recovered within 3 months.

An interesting observation from our study is that the worst AVQI value was recorded in the sole patient who experienced postoperative bleeding, possibly due to higher morbidity associated with revision surgery or laryngeal mechanical injury from second oro-tracheal intubation. AVQI values from patients with vocal fold impairment normalized later than endoscopic findings, suggesting residual functional deficits that may only be identified through voice frequency analysis.

In our study, about 50% of patients presented with AVQI values higher than 2.35 at baseline, indicating the presence of hoarseness preoperatively. This observation may be attributed to vocal cord dysfunction related to patient anxiety in the immediate preoperative period. Our findings are consistent with other studies showing a significant proportion of patients with AVQI values above the pathological threshold preoperatively [16,18,19]. The same authors emphasize the importance of considering the variation (delta) rather than the absolute value.

Additionally, our study aimed to investigate a potential correlation between vocal outcomes and certain variables (gender, age, and central neck dissection) that might be linked to a higher risk of vocal impairment [20]. In our study population, gender and central neck dissection do not appear to significantly affect the Acoustic Voice Quality Index (AVQI) values. However, younger patients seem to experience worse vocal outcomes compared to older patients, although this difference tends to diminish over time but remains noticeable even after one month. The explanation could be that younger patients started with higher baseline values due to a significant proportion of them being active smokers (73%). Additionally, since most of them were voice professionals (64%), premature vocal strain during the recovery phase may have led to poorer AVQI outcomes. Finally, the different group sizes might also have influenced the results obtained. Our findings are supported by other studies that found no relationship between AVQI value and gender or age [20,21], although data regarding central neck dissection in the literature are still conflicting. Among patients with post-surgical vocal fold impairment, 80% were affected by larger hyperfunctioning nodules, which are associated with more challenging thyroid dissections.

Considering the very low incidence of vocal cord impairment after thyroid surgery in our study, the real effect of early speech rehabilitation could be understood but could not demonstrated on strong evidence. However, everyone improved by undergoing early speech therapy.

A key limitation of our study is the small sample size. Conducting a larger multi-center prospective study could mitigate this limitation and provide more reliable information about the influence of various factors on post-thyroidectomy vocal outcomes.

Another possible limit is that a subjective evaluation of the voice has not been considered.

An intriguing future study could involve revision surgery cases were in our experience (only one case) AVQI worsen more.

In conclusion, our study did not find a significant worsening of vocal performance measured by AVQI in patients undergoing thyroidectomy for DTC. Age, gender, and central neck dissection do not appear to be significantly associated with a higher risk of vocal changes. Future multi-center prospective studies with larger sample sizes are warranted to validate these findings and explore potential correlations between subjective voice evaluations and AVQI values.

## Figures and Tables

**Table 1:** Italian Praat sentences.

	Italian	English
1	Il nuovo libro verde è sulla scatola	The new green book is on the box
2	L’uomo e la donna mangiano le uova	The man and the woman eat eggs
3	Che cosa ha rotto il gatto?	What broke the cat?
4	Le mie nonne non vanno mai al mare	My grandmothers never go to the beach
5	Lo zoppo ha toccato il letto	The lame man touched the bed

**Table 2:** Characteristics of patients, surgery procedure, staging, and complications.

Surgery		N (%)
Total Thyroidectomy		63 (85.13%)
Lobo isthmectomy		11 (14.87%)

**Histology**		**N (%)**
Papillary carcinoma		61 (82.43%)
Follicular carcinoma		5 (6.67%)
Mixed papillary/follicular carcinoma		8 (10.81%)

**T stage at diagnosis according to AJCC TNM**		**N (%)**
T1	a	43 (58.11%)
	b	13 (17.57%)
T2		12 (16.21%)
T3		6 (8.11%)
T4		0

		**N (%)**
**Incidental diagnosis**		19 (25.68%)

**Central compartment neck dissection**		**N (%)**
No neck dissection		45 (60.81%)
Central neck dissection		29 (39.19%)
	Metastasis +	13 (44.83%)
	Metastasis −	16 (55.17%)

**Post operative complications**		
Transient hypocalcemia (Ca serum level < 8.5 mg/dL)		24 (32.42%)
Bleeding		1 (0.74%)

**Table 3:** Data of the 5 patients affected by vocal cord impairment after surgery. Normal values: green, altered values: red.

	Preadmission		T1 (10 Days)		T2 (1 Month)		T3 (3 Months)	
	Vocal Cord Motility	AVQI Value	Vocal Cord Motility	AVQI Value	Vocal Cord Motility	AVQI Value	Vocal Cord Motility	AVQI Value
Patient 1	Normal	1.97	Impaired	5.68	Impaired	5.24	Normal	2.14
Patient 2	Normal	2.45	Impaired	3.81	Normal	2.33	Normal	2.76
Patient 3	Normal	2.01	Impaired	3.52	Normal	2.24	Normal	2.10
Patient 4	Normal	3.97	Impaired	8.62	Impaired	7.75	Normal	2.45
Patient 5	Normal	1.98	Impaired	4.42	Normal	4.59	Normal	1.83

## Data Availability

All data are available on request.
